# Traditional Chinese medicine interventions for post-stroke emotional disorders: a protocol for network meta-analysis of randomized controlled trials

**DOI:** 10.3389/fpsyt.2026.1648067

**Published:** 2026-02-18

**Authors:** Yuqiao Chao, Bai Li, Zhaozhan Xie, Hongling Jia, Yongchen Zhang

**Affiliations:** 1 The Second Affiliated Hospital of Shandong University of Traditional Chinese Medicine, Jinan, China; 2 College of Acupuncture and Tuina, Shandong University of Traditional Chinese Medicine, Jinan, China; 3 Second School of Clinical Medicine, Shandong University of Traditional Chinese Medicine, Jinan, China

**Keywords:** network meta-analysis, post-stroke emotional disorders, study protocol, systematic review, traditional Chinese medicine

## Abstract

**Background:**

Post-stroke emotional disorders (PSED), primarily encompassing depression, anxiety, and their comorbid conditions, are common and serious complications of stroke. PSED has a high prevalence, significantly reduces patients’ quality of life, impedes neurofunctional recovery, and increases the risk of stroke recurrence and mortality. While modern medical treatments are effective, they are generally limited by slow onset, suboptimal response rates, and side effects, and often target a single pathological mechanism, making them inadequate to address the complex interplay of multiple mechanisms characteristic of PSED. Traditional Chinese Medicine (TCM) demonstrates potential advantages through multi-target regulation and relatively good safety. However, the relative efficacy ranking of its numerous therapeutic approaches still lacks robust evidence-based support.

**Methods:**

This study plans to conduct a network meta-analysis (NMA), systematically searching multiple Chinese and English databases and grey literature since the 21st century, including PubMed, Embase, Cochrane CENTRAL, Web of Science, CNKI, CBM, VIP, Wanfang, and ClinicalTrials. Randomized controlled trials (RCTs) comparing different TCM therapies with conventional Western medicine or placebo control for adult PSED will be included. The primary outcome measures include the total clinical effective rate and changes in standardized scale scores. Study screening, data extraction, and risk of bias assessment will be independently completed by two researchers, and any discrepancies will be resolved through discussion or third-party arbitration. A random-effects NMA model based on the Bayesian statistical framework will be constructed to quantify the efficacy differences among different TCM interventions. Additionally, subgroup analyses will be conducted based on PSED types and TCM syndrome patterns to further explore the heterogeneity of efficacy. This study will also comprehensively evaluate the transitivity assumption, heterogeneity, and inconsistency, and use the GRADE framework to grade the quality of evidence. The study protocol strictly follows the PRISMA-P and NMA guidelines and has been registered on the PROSPERO platform.

**Results:**

This NMA will synthesize direct and indirect evidence to quantify effect sizes and rank probabilities of diverse TCM interventions for PSED. Key outputs will include:1. Pairwise comparisons among TCM interventions and between each TCM intervention versus control groups; 2. The ranking of the efficacy of each TCM intervention measure in improving the symptoms of PSED; 3. Subgroup analyses of treatment effects stratified by PSED subtypes and TCM syndrome patterns; 4. Evidence certainty ratings for all effect estimates using the GRADE framework.

**Conclusion:**

This study expects to rigorously conduct a network meta-analysis that will, for the first time within the TCM syndrome differentiation and treatment framework, systematically compare the relative efficacy of multiple TCM therapies for PSED, quantifying their effect sizes and optimal ranking probabilities. The results will provide clinicians with evidence-based hierarchical selection criteria for “acupuncture-Chinese herbal medicine-integrated therapy”, addressing the current evidence gap in TCM-based decision-making for PSED. This protocol emphasizes the critical importance of subgroup analyses by PSED subtypes and TCM syndrome patterns, aiming to establish the foundation for more precise and individualized integrated Chinese-Western treatment regimens. Public preregistration enhances research transparency and result credibility, promotes the production of high-quality TCM evidence and its international recognition, ultimately serving to optimize clinical management and improve prognosis in PSED patients.

**Systematic Review Registration:**

https://www.crd.york.ac.uk/prospero/, identifier CRD420251069356.

## 1 Introduction

Post-stroke emotional disorders (PSED) represent prevalent neuropsychiatric complications following stroke, predominantly manifesting as depression, anxiety, and their comorbid conditions (1). Epidemiological studies indicate their prevalence correlates with time course and gender; specifically, the 1-year aggregate prevalence of post-stroke anxiety (PSA) approximates 29.3% (2), while post-stroke depression (PSD) exhibits a 2-year incidence ranging from 11% to 41% (3). PSED not only significantly diminishes patients’ quality of life but also impedes neurological rehabilitation, exacerbates cognitive impairment, and reduces treatment adherence. These effects substantially increase stroke recurrence risk and mortality (4), thereby establishing a vicious cycle of “brain injury → emotional dysfunction → clinical deterioration”. This complex pathophysiology underscores the imperative for comprehensive exploration of pathogenic mechanisms and targeted interventions.

Based on the aforementioned clinical risks, modern medical research has revealed that the pathogenesis of PSED involves synergistic interactions among multiple mechanisms (5). Stroke directly damages monoaminergic pathways in the brainstem, such as the raphe nuclei and locus coeruleus, leading to reduced synthesis of serotonin (5-HT), norepinephrine (NE), and dopamine (DA). This impairs patients’ ability to regulate emotions (6). Additionally, ischemia and hypoxia trigger massive glutamate release, which induces calcium overload and excitotoxicity through NMDA and AMPA receptors. This ultimately results in neuronal death in the prefrontal cortex and limbic system, while suppressing the production of brain-derived neurotrophic factor (BDNF) (7–9).This process further activates microglia, prompting the release of pro-inflammatory cytokines such as IL-1β and TNF-α. These cytokines not only directly inhibit monoamine synthase activity but also increase cortisol secretion by activating the hypothalamic-pituitary-adrenal (HPA) axis, thereby accelerating monoamine neurotransmitter depletion (10). Simultaneously, stroke-induced sympathetic hyperactivity causes intestinal ischemia and gut dysbiosis. Metabolic products from pathogenic bacteria enter the brain via the circulatory system and vagus nerve, exacerbating central inflammation and reducing the conversion of tryptophan to 5-HT (11). Infiltration of peripheral neutrophils releases matrix metalloproteinase-9 (MMP-9), which disrupts the blood-brain barrier and facilitates the spread of inflammation within the brain. The resulting burst of reactive oxygen species (ROS) forms a positive feedback loop with glutamate excitotoxicity. Together, these factors suppress hippocampal neurogenesis and synaptic plasticity. These mechanisms are interlinked: glutamate excitotoxicity exacerbates oxidative stress; inflammation aggravates monoamine imbalance; dysregulation of the gut-brain axis and systemic inflammation mutually reinforce each other. Ultimately, this cascade leads to functional collapse of the prefrontal-limbic network, driving persistent worsening of emotional disorder symptoms. Currently, pharmacological treatments for PSED include monoaminergic system modulators, glutamatergic system modulators, and gamma-aminobutyric acid (GABA) system modulators (5, 12). Monoaminergic system modulators encompass drugs with two distinct mechanisms: one involves monoamine reuptake inhibitors (SSRIs, SNRIs, and TCAs) that directly elevate monoamine levels in the synaptic cleft by blocking 5-HT/NE reuptake, thereby improving mood and motivation (13, 14); the other comprises noradrenergic and specific serotonergic antidepressants (NaSSAs) that selectively block α_2_-adrenergic receptors along with 5-HT_2_ and 5-HT_3_ receptors, promoting NE and 5-HT release while activating 5-HT_1_A receptors (15). Both ultimately increase brain monoamine neurotransmitter levels. However, SSRIs and SNRIs exhibit limitations including delayed onset, suboptimal response rates, and potential increased risk of cerebral hemorrhage (16, 17). Compared to SSRIs, TCAs demonstrate higher adverse reaction rates in elderly patients and may cause cardiac suppression, resulting in lower safety; consequently, they are no longer recommended as first-line therapeutics (18). Although NaSSAs are not associated with severe adverse reactions, they tend to induce sedative effects, and concomitant use with other sedative-hypnotic agents should be avoided. Glutamatergic system modulators can rapidly alleviate depressive symptoms in treatment-resistant post-stroke depression(PSD) by blocking NMDA receptors (19), but their clinical application is substantially limited by risks of hallucinogenic effects, addiction potential, and long-term neurotoxicity. Gamma-aminobutyric acid (GABA) system modulators, specifically benzodiazepines (BZDs), function as positive allosteric modulators of GABA_A_ receptors. They enhance endogenous GABA binding efficacy, promoting chloride channel opening and influx, which induces neuronal hyperpolarization and inhibition. This effect is particularly pronounced in key anxiety-regulating brain regions such as the amygdala, where they mediate rapid anxiolytic effects by suppressing neuronal hyperexcitability. Prolonged use, however, may cause dependence, tolerance, and withdrawal symptoms. A systematic review further highlighted BZDs’ association with elevated overall mortality risk (20). Although numerous pharmacological options exist, all exhibit significant side effect limitations, and crucially, current drugs predominantly target single mechanistic pathways, failing to address PSED’s multi-mechanistic pathophysiology.

Within this context, TCM demonstrates multi-pathway regulatory advantages through comprehensive therapies including compound formulations, acupuncture, and other treatment modalities. Research indicates that Chinese herbal compounds and acupuncture therapy can ameliorate PSED via multiple mechanisms: regulating neurotransmitters, mitigating neuronal damage, suppressing hypothalamic-pituitary-adrenal (HPA) axis hyperactivity, reducing immune-inflammatory responses, and modulating gut microbiota. Studies have shown that the “Tongdu Tiaoshen(Unblocking Governor Vessel and Regulating Mind)” acupuncture technique, primarily targeting acupoints Baihui (GV20), Shuigou (GV26), Shenting (GV24), and Dazhui (GV14), effectively alleviates ultrastructural damage in the hippocampal CA1 region of post-stroke depression(PSD) model rats, and this intervention increases hippocampal levels of neurotransmitters NE, 5-HT, and DA while improving depressive-like behaviors (21). Electroacupuncture elevates hippocampal expression of BDNF, TrkB, and cAMP response element-binding protein (CREB) in PSD model rats, enhancing synaptic activity (22). Acupuncture significantly reduces serum levels of CRH, ACTH, and CORT in PSED patients, correcting HPA axis dysregulation and exerting anxiolytic and antidepressant effects (23). Electroacupuncture at Baihui (GV20), Shenting (GV24), Zusanli (ST36), and Yanglingquan (GB34) downregulates serum pro-inflammatory cytokines IL-1β, IL-6, and TNF-α, attenuating inflammatory responses and promoting hippocampal neural repair (24, 25). Sun demonstrated that “Tiaoshen Yunshu(Mind-Regulating and Pivot-Moving)” acupuncture increases the relative abundance of Lactobacillus and Bifidobacterium while reducing Escherichia coli and Enterococcus in PSD patients, and this technique upregulates beneficial microbial metabolites SCFAs, elevates neuroprotective gut-derived butyrate concentrations, ameliorates cerebral neuroinflammation, and effectively alleviates depressive states (26). Regarding Chinese herbal compounds, studies reveal that *Si-Ni-San* inhibits excessive NMDAR activation in the dorsal raphe nucleus (DRN), balancing 5-HTergic and GABAergic neuronal activity to improve anxiety-depression-like behaviors in adolescent mice (27). *Xiao-Yao-San* bidirectionally regulates gene expression in the hippocampus and amygdala, modulates cerebral neurotransmitters including DA, NE, and 5-HT, inhibits M1 polarization of hippocampal microglia to exert anti-inflammatory effects, and promotes hippocampal neurogenesis (28). Additionally, multiple compound formulations such as *Gui-Pi-Tang*, *Chaihu-Shugan-San*, and *Xingnao Jieyu* Capsules demonstrate efficacy in ameliorating negative emotions including anxiety and depression. For non-pharmacological and non-invasive therapies like music intervention, clinical studies indicate that the “Jue-note” in Five-Element Music therapy soothes liver stagnation, regulates emotional states, and improves heart rate variability to enhance autonomic nervous function. This intervention regulates qi-blood dynamics, reduces serum cortisol levels, and alleviates anxiety symptoms. Furthermore, Jue-note music increases serum superoxide dismutase (SOD) while decreasing malondialdehyde (MDA) levels, exerting antioxidant effects that improve depressive symptoms and sleep disorders (29).

Notably, although TCM demonstrates multi-target regulatory advantages against PSED’s complex pathophysiology and numerous clinical observations indicate its overall adverse reaction rate is generally lower than that of Western pharmaceuticals (30, 31), hierarchical efficacy ranking among TCM therapies remains unsupported by high-level evidence due to therapeutic diversity and the TCM-specific paradigm of syndrome differentiation-based treatment. To address this critical gap, NMA serves as a quantitative methodology capable of globally evaluating comparative effectiveness across multiple interventions by synthesizing direct and indirect evidence (32).Within this study context, while head-to-head clinical trials comparing different TCM therapies are lacking, NMA enables indirect comparisons when pairwise interventions share common comparator arms, thereby integrating all therapies within a unified effect size framework. This indirect evidence-based approach has become essential for evidence synthesis and clinical decision-making in healthcare (33), particularly valuable for overcoming limitations of traditional direct comparisons—especially when clinicians require identification of the “optimal single therapy” from multiple treatment options (32).Through NMA, systematic comparisons of effect sizes among acupuncture, Chinese herbal medicine, and other interventions—while preserving TCM’s syndrome differentiation principle—can establish an evidence-based decision-making framework for developing hierarchical integrated Chinese-Western therapeutic protocols.

## 2 Materials and methods

This study rigorously adheres to the PRISMA-P guidelines for framework development and will comply with PRISMA-NMA standardized reporting requirements during the results presentation phase (34, 35) (**Supplementary Files 1, 2**). To further enhance the decision-support value of the research, we incorporate the Indirect Treatment Comparison/Network Meta-Analysis Study Questionnaire issued by the International Society for Pharmacoeconomics and Outcomes Research (ISPOR). This tool systematically evaluates clinical relevance, methodological credibility, and evidence translation efficiency to improve evidence interpretation capabilities for clinicians and health policy-makers (36). The study protocol has been prospectively registered on the PROSPERO international platform for systematic reviews (Registration ID: CRD420251069356). All protocol amendments will be updated in real-time through the platform’s version control functionality, ensuring transparent and traceable research conduct.

### 2.1 Search strategy

This study will systematically search the Cochrane Central Register of Controlled Trials (CENTRAL), Embase, PubMed, Web of Science, and four major Chinese databases(China National Knowledge Infrastructure (CNKI), Chinese Biomedical Literature Database (CBM), VIP Chinese Science and Technology Journal Database (VIP), and Wanfang Database). Additionally, we will search the ClinicalTrials.gov registry for unpublished complete clinical trial data, while extending to grey literature, international conference proceedings, and reference lists of included studies and existing systematic reviews. To ensure evidence completeness, corresponding authors and principal investigators will be proactively contacted to supplement incompletely reported data in primary studies or obtain raw data from unpublished research. This strategy aims to minimize publication bias and construct a comprehensive evidence network. All randomized controlled trials (RCTs) published since the inception of the 21st century (January 1, 2000, onward) investigating TCM interventions for post-stroke emotional disorders will be comprehensively collected. The search strategy employs a combination of Medical Subject Headings (MeSH) and free-text terms, with search syntax adapted to each database’s indexing rules and controlled vocabularies. For English databases, using PubMed as an example, a structured search syntax was constructed using Boolean operators; the specific search strategy is detailed in **Table 1**. For Chinese databases, using CNKI as an example, its search strategy is shown in **Table 2**. To ensure accurate correspondence between Chinese and English search terms, bilingual researchers familiar with the field verified and refined the candidate terms with reference to the *Chinese Medical Subject Headings (*37) and the built-in subject heading systems of databases such as CBM and CNKI, to identify the most precise and commonly used controlled vocabulary. Subsequently, another independent reviewer back-translated the finalized Chinese subject terms into English and compared them with the original English terms to ensure conceptual consistency and minimize semantic bias. The search strategies for other Chinese databases were adapted based on the CNKI search strategy, modifying the syntax according to the requirements of each database’s search engine while keeping the core set of search terms unchanged.

**Table 1:** Search strategy for the PubMed database.

Number	Search items
#1	Stroke[MeSH Terms] OR Stroke[Title/Abstract] OR Cerebrovascular Accident[Title/Abstract] OR CVA[Title/Abstract]
#2	Mood Disorders[MeSH Terms] OR Mood Disorders[Title/Abstract] OR Emotional Disorders[Title/Abstract] OR Affective Disorders[Title/Abstract]
#3	Post-Stroke Depression[MeSH Terms] OR (Post-Stroke Depression) OR (Depression after Stroke)
#4	Post-Stroke Anxiety[MeSH Terms] OR (Post-Stroke Anxiety) OR (Anxiety after Stroke)
#5	Acupuncture[MeSH Terms] OR Acupuncture[Title/Abstract] OR Needle Therapy[Title/Abstract] OR Acupressure[Title/Abstract] OR Acupoint Stimulation[Title/Abstract]
#6	Traditional Chinese Medicine[MeSH Terms] OR Traditional Chinese Medicine[Title/Abstract] OR TCM[Title/Abstract] OR Herbal Medicine[Title/Abstract] OR Chinese Herbal Medicine[Title/Abstract]
#7	Massage[MeSH Terms] OR Massage[Title/Abstract] OR Manipulative Therapy[Title/Abstract] OR Manual Therapy[Title/Abstract] OR Bodywork[Title/Abstract]
#8	Traditional Exercises[Title/Abstract] OR (Traditional Exercises) OR (Qigong) OR (Tai Chi)
#9	Randomized Controlled Trial[MeSH Terms] OR Randomized Controlled Trial[Title/Abstract] OR RCT[Title/Abstract] OR Randomized Study[Title/Abstract] OR Clinical Trial[Title/Abstract]
#10	#2 OR #3 OR #4
#11	#5 OR #6 OR #7 OR #8
#12	#1 AND #10 AND #11

**Table 2:** CNKI search strategy.

Number	Search items	Logic	Search field
#1	Stroke + Apoplexy + Cerebrovascular Accident	OR	Subject
#2	Mood Disorders + Emotional Disorders + Depression + Anxiety + Post-Stroke Depression + Post-Stroke Anxiety	OR	Subject
#3	Traditional Chinese Medicine + Traditional Chinese Medicine (TCM) + Chinese Herbal Medicine + Acupuncture + Electroacupuncture + Scalp Acupuncture + Needling + Qigong + Tai Chi + Baduanjin + Five-Element Music	OR	Subject
#4	Randomized Controlled Trial + RCT + Randomized Control + Clinical Controlled Trial	OR	Subject
#5	#1 AND #2 AND #3 AND #4	AND	

### 2.2 Eligibility criteria

#### 2.2.1 Inclusion criteria

1) Study Population

Diagnostic Criteria: Stroke diagnosis must comply with the 2024 AHA/ASA Guidelines for the Primary Prevention of Stroke (38) or the Chinese Guidelines for Cerebrovascular Disease Prevention and Treatment (2024) (39).

Emotional Disorder Diagnosis: Depression: Meets DSM-5/ICD-10 criteria for depressive episode, with SDS score ≥ 53 or HAMD-17 score ≥ 8; Anxiety: GAD-7 score ≥5 or HAMA score ≥ 7; Mixed Type: Concurrent depressive and anxiety symptoms with HADS mean score ≥ 8.

Disease Course: Emotional disorders emerging between 2 weeks and 1 year post-stroke.

Age: 18–75 years, no gender restrictions.

2) Interventions

TCM Intervention Types: Chinese Herbal Medicine: Compound formulas; single herbs; proprietary Chinese medicines;

Acupuncture: Body acupuncture; scalp acupuncture; electroacupuncture;

Other Therapies: Qigong; Tai Chi; Baduanjin; Five-Element Music therapy;

Combination Therapies: TCM integrated with Western medicine; multimodal TCM approaches.

Intervention Details: Treatment frequency and duration must be reported; herbal formulas require specification of decoction methods and dosages.

3) Comparator Groups

Control Types:

Active Control: Conventional Western therapy (e.g., pharmacotherapy, cognitive behavioral therapy).

Negative Control: Placebo (e.g., sham acupuncture, herbal placebo).

No-Intervention Control: Basic stroke care only (e.g., aspirin, rehabilitation).

Permitted Co-Interventions: Control groups may receive foundational stroke treatments (e.g., antiplatelet agents).

4) Outcome Measures

Primary Outcomes:

Clinical Response Rate: Total effective rate; (To control for heterogeneity, only studies that explicitly define the total effective rate will be included. The definition must be based on standardized scales recognized in this study and must clearly report the thresholds for “marked improvement” and “improvement.” Studies using self-defined criteria, vague definitions, or failing to report the specific calculation method will be excluded).

Standardized Scale Scores: Pre-post treatment differences in HAMD-17, HAMA, SDS, GAD-7, or HADS scores.

Secondary Outcomes:

Quality of Life: Changes in scores assessed by standardized QoL instruments, including but not limited to the 36-Item Short Form Health Survey (SF-36), the Stroke-Specific Quality of Life Scale (SS-QOL), or the WHO Quality of Life-BREF (WHOQOL-BREF).

5) Study Design

Design: Parallel-group randomized controlled trials (RCTs) with intervention duration ≥4 weeks.

Sample Size: ≥20 participants per group to minimize bias.

Publication Period: Studies published between January 2000 and May 2025 (excludes methodologically immature early studies and obsolete therapies).

Language: Chinese or English publications.

#### 2.2.2 Exclusion criteria

1) Study Population

Severe cognitive impairment or aphasia precluding assessment cooperation;History of psychiatric disorders (e.g., schizophrenia, bipolar affective disorder);Substance abuse (e.g., alcohol dependence);Pre-existing unresolved emotional disorders prior to stroke onset (to avoid confounding efficacy evaluation).

2) Interventions

Non-traditional TCM methods (e.g., infrared “acupuncture,” unvalidated device-based therapies);Interventions lacking TCM theoretical basis (e.g., proprietary Chinese medicines administered without syndrome differentiation).

3) Outcome Measures (Outcomes)

Sole use of non-validated or self-designed assessment tools; Failure to distinguish stroke-related emotional disorders from other complications (e.g., fatigue, pain) in outcome reporting.

4) Study Design

Non-controlled studies; Duplicate publications or inaccessible full texts.

### 2.3 Data collection and analysis

#### 2.3.1 Study selection

**Figure 1:**
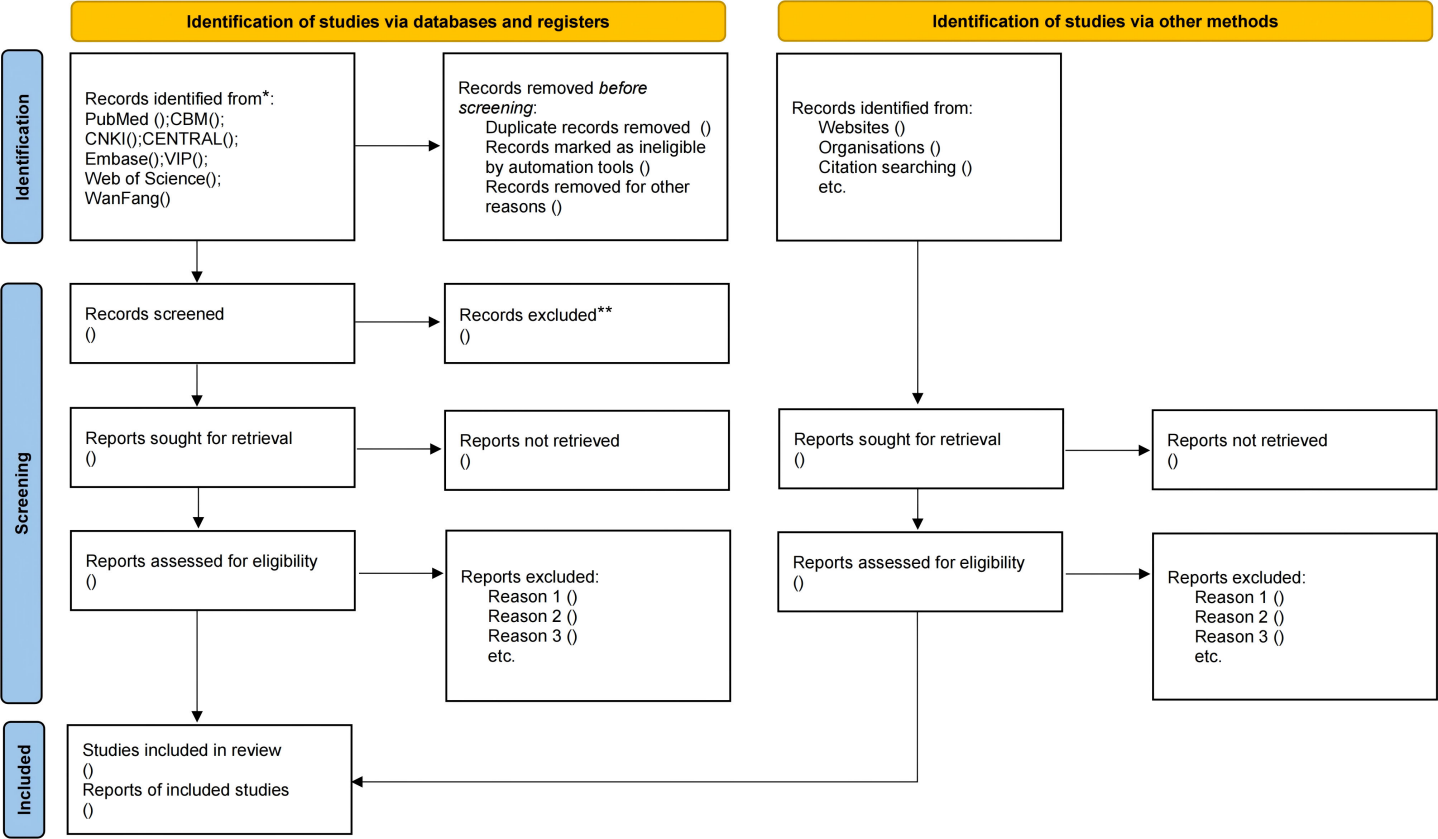
PRISMA flow chart of study selection process. *Consider, if feasible to do so, reporting the number of records identified from each database or register searched (rather than the total number across aall databases/registers). **If automation tools were used, indicate how many records were excluded by a human and how many were excluded by automation tools.

This study will implement a standardized literature management workflow: Retrieved citations will undergo automated and manual deduplication in EndNote 2025, with the deduplicated library subsequently uploaded to the Covidence platform (40). Within this system, two independent reviewers will conduct preliminary title/abstract screening for all records; a randomly selected 10% subset will undergo dual independent screening to quantify inter-rater agreement via Kappa statistics (target κ≥0.8), with discrepancies resolved through consensus discussion, while the remaining records will be screened independently. Studies passing initial screening will proceed to full-text assessment, wherein corresponding authors of publications with incomplete data will be contacted for supplementation; all exclusion decisions will be categorically documented in Covidence per PRISMA-P standards, automatically generating auditable exclusion records. The platform’s built-in functionality will ultimately export a PRISMA 2020-compliant flow diagram to dynamically visualize the screening process (41) (provisional diagram: **Figure 1**). For data extraction, customized Covidence electronic forms will facilitate dual independent data entry, automatically flagging discordant entries for arbitration until consensus is achieved, ensuring dataset accuracy and methodological transparency.

#### 2.3.2 Data extraction process

Dual Independent Extraction: Two reviewers will independently extract data using a structured extraction template encompassing:

Study Characteristics: Authors, publication year, journal, country, funding sources.

Design Information: Intervention types, comparator groups, total study duration, diagnostic criteria.

Sample Characteristics: Sample size per group, age/gender/racial composition.

Outcome Metrics: Baseline and post-intervention scale scores (HAMD-17, HAMA, SDS, GAD-7, HADS, SF-36, SS-QOL, WHOQOL-BREF).

TCM Syndrome Patterns: With reference to the TCM syndrome classification in the Guidelines for the Integrated Clinical Diagnosis and Treatment of Mental Disorders - Post-Stroke Mental Disorders (42), a standardized syndrome reference table will be developed to serve as the basis for data extraction and subsequent subgroup analysis, as shown in **Table 3**.

**Table 3:** Diagnostic criteria for traditional chinese medicine (TCM) syndrome patterns.

TCM syndrome pattern	Diagnostic criteria
Gan Yu Qi Zhi Zheng (Liver Qi Stagnation)	**Core symptoms:** Mental depression, emotional unrest; Distending pain in chest and hypochondrium (no fixed location); Epigastric stuffiness and belching **Secondary symptoms:** Anorexia, irregular defecation; Thin greasy tongue coating; Wiry pulse (Xian Mai) **Key Points for Syndrome Differentiation:** Meets ≥2 core symptoms + ≥1 secondary symptom
Qi Yu Hua Huo Zheng (Qi Stagnation Transforming into Fire)	**Core symptoms:** Sudden facial paralysis, slurred speech, limb clumsiness (even hemiplegia); Irritability, easy anger; Distending pain in chest and hypochondrium; Bitter and dry taste in mouth **Secondary symptoms:** Headache, red eyes, tinnitus; Acid regurgitation, constipation; Red tongue with yellow coating; Rapid wiry pulse (Xian Shu Mai) **Key Points for Syndrome Differentiation:** Meets ≥2 core symptoms + ≥1 secondary symptom
Tan Meng Qing Qiao Zheng (Phlegm Obscuring the Clear Orifices)	**Core symptoms:** Sudden syncope, unconsciousness;Locked jaw, clenched fists, limb spasticity; Absence of defecation and urination; Pale complexion, dark lips **Secondary symptoms:** Mental trance, poor memory, drowsiness; Dizziness, cold limbs; Profuse phlegm; Dark tongue with white greasy coating; Deep slippery pulse (Chen Hua Mai) **Key Points for Syndrome Differentiation:** Meets ≥2 core symptoms + ≥1 secondary symptom
Xin Pi Liang Xu Zheng (Heart-Spleen Deficiency)	**Core symptoms:** Excessive worrying, mental restlessness; Palpitations, timidity- Dizziness, fatigue **Secondary symptoms:** Insomnia, amnesia; Pale complexion, poor appetite; Pale tongue with thin white coating; Thin weak pulse (Xi Ruo Mai) **Key Points for Syndrome Differentiation:** Meets ≥2 core symptoms + ≥1 secondary symptom
Gan Shen Yin Xu Zheng (Liver-Kidney Yin Deficiency)	**Core symptoms:** Emotional unrest, palpitations, amnesia; Dizziness, tinnitus, soreness of waist and knees **Secondary symptoms:** Insomnia, dreaminess; Five-center heat (palms, soles, chest), night sweats; Dry mouth and throat; Red tongue with little fluid; Thin weak or deep thin pulse (Xi Ruo/Chen Xi Mai) **Key Points for Syndrome Differentiation:** Meets ≥2 core symptoms + ≥1 secondary symptom
Yu Xue Nei Zu Zheng (Blood Stasis Blockage)	**Core symptoms:** Mental depression, irritability; Headache, insomnia, amnesia;Chest and hypochondrium pain, Local cold or heat sensation in body **Secondary symptoms:** Purple dark tongue (with ecchymoses/petechiae); Wiry or unsmooth pulse (Xian/Se Mai) **Key Points for Syndrome Differentiation:** Meets ≥2 core symptoms + ≥1 secondary symptom

Bold text includes three parts: 1) the description of core diagnostic criteria (Core symptoms); 2) the description of secondary diagnostic criteria (Secondary symptoms); 3) the syndrome differentiation rules (Key Points for Syndrome Differentiation). Among them, the core symptoms are the key indicators to distinguish different syndrome types and determine the syndrome differentiation result.

Follow-up Data: Scale scores categorized by endpoint: short-term (≤90 days), mid-term (91–364 days), and long-term (>364 days).

Consistency Validation: Inter-rater agreement will be quantified using the Kappa statistic (κ≥0.8 required). If the naming of syndrome patterns in the included studies varies, two TCM clinical experts will perform a consensus classification based on the above diagnostic criteria, preferentially assigning them to the syndrome type with the highest match to core symptoms. Studies that cannot be clearly classified will be included in an “other syndrome patterns” subgroup or excluded. In case of any other disagreements, a third reviewer will arbitrate to reach a consensus.

#### 2.3.3 Data management and export

All extracted data will undergo cross-verification and organization within the Covidence literature management platform, with final exportation in structured.csv format to support subsequent statistical analysis.

#### 2.3.4 Intervention group classification

Intervention group classification will be completed during the data extraction phase. The intervention groups in each trial will be categorized according to the following classes: the TCM interventions will be classified as follows: Chinese herbal compound formulas with clearly defined compositions and syndrome differentiation basis; acupuncture therapies conforming to the STRICTA reporting guidelines (43); integrated TCM therapies; and other TCM therapies. Control condition types are classified as: conventional Western medicine treatment, where the categories of anti-affective disorder drugs included in this study are based on evidence-based therapeutic agents recommended by the American Psychiatric Association medication guidelines (44), including selective serotonin reuptake inhibitors (SSRIs, e.g., sertraline, fluoxetine), serotonin-norepinephrine reuptake inhibitors (SNRIs, e.g., venlafaxine), and tricyclic antidepressants (TCAs, e.g., amitriptyline), etc.; placebo control; and no-treatment control. If classification discrepancies cannot be resolved by a third-party reviewer, consultation will be sought with the project advisory group, comprising TCM clinical experts and psychiatric specialists, to determine the final classification through consensus.

This study will generate a network plot to visualize the available evidence structure. This plot will depict all possible direct comparisons between interventions, with the thickness of the connecting lines between nodes, or numerical annotations, reflecting the number of included studies or sample size for each comparison pair. The reference node will be set as the active control condition, and different intervention combinations will be presented as distinct nodes (45). This figure will intuitively display the connectivity of the evidence network and the comparison pathways, providing a topological basis for subsequent statistical analysis. To address the potential issue of sparse network connectivity resulting from overly granular classification, we will adhere to the principle of “clinical similarity first.” Based on clinical criteria of “similarity in therapeutic principle” and “similarity in herbal composition or acupoint selection,” two reviewers will independently assess and subsequently submit cases for arbitration by an expert advisory panel to merge similar interventions into a single node.

#### 2.3.4 Risk of bias assessment

Studies passing full-text screening will undergo risk of bias assessment. This assessment will be independently performed by two reviewers using the Cochrane Risk of Bias tool on the Covidence platform (46). The tool assesses key domains including the method of random sequence generation, allocation concealment, blinding of participants and personnel, blinding of outcome assessors, completeness of outcome data, and risk of selective outcome reporting.

#### 2.3.5 Summary measures

The summary measures for this study will report scores for anxiety, depression, comorbid conditions and quality of life. For studies employing continuous data, the effect size between treatment groups will be presented as the mean difference (MD). The mean difference will be calculated as the follow-up score minus the baseline score (with some studies directly reporting the difference). Any study unable to provide any two of the following—baseline data, change values, or follow-up data—will be excluded. Furthermore, studies failing to report the standard deviation (SD) of the score change or the follow-up SD (or where this cannot be calculated from confidence intervals or standard errors) will also be excluded. These criteria aim to reduce the risk of bias while maintaining the informational integrity of the evidence network. Data will also be summarized via treatment rankings and the surface under the cumulative ranking curve (SUCRA) to aid in interpreting the relative effectiveness of all intervention types within the network.

#### 2.3.6 Transitivity assessment

To validate the effectiveness of indirect comparisons within the network meta-analysis (NMA) and ensure the scientific validity and clinical plausibility of comparative results across different interventions, a table of trial characteristics that may act as effect modifiers will be compiled from the collected data. This table will include: ① Stroke-related factors (severity, type); ② PSED-related factors (time of onset, subtype); ③ Patient-related factors (age, sex, cognitive function); and ④ Co-interventions. By analyzing whether the distribution of potential effect modifiers is balanced across studies, this approach will assess whether these factors introduce heterogeneity that interferes with treatment effects, thereby supporting the evaluation of the transitivity assumption (47, 48). If clinically significant imbalances in key factors are identified, this will be explicitly noted in the interpretation of indirect comparisons and will serve as the basis for conducting pre-planned meta-regression or subgroup analyses.

#### 2.3.7 Data synthesis

The primary objective of data synthesis in this study is to compare the efficacy differences among different Traditional Chinese Medicine (TCM) therapies. As network meta-analysis (NMA) can compensate for the lack of direct evidence through indirect estimations, it is suitable for achieving this objective. Currently, no head-to-head clinical trials have been conducted between distinct TCM therapies; therefore, studies will be pooled for analysis according to the intervention group classifications defined above. If quantitative synthesis of study results is infeasible, findings will be presented through a descriptive systematic review. All quantitative analyses will be performed using Stata 18 and RStudio software.

#### 2.3.8 Pairwise meta-analysis

If included studies provide direct comparison data between different Traditional Chinese Medicine (TCM) interventions, an exploratory pairwise meta-analysis will be performed. A random-effects model will be employed for analysis, with forest plots used to display individual study results and pooled effect sizes; separate independent analyses will be conducted for each primary outcome measure. Publication bias and small-study effects will be assessed using funnel plots and Egger’s regression test. If significant bias is detected (Egger’s test *P* < 0.10), the trim-and-fill method will be applied to adjust effect size estimates (49).

#### 2.3.9 Subgroup analysis

Given that this study examines the effectiveness of diverse Traditional Chinese Medicine (TCM) interventions for post-stroke emotional disorders (PSED), subgroup analyses will be implemented as follows: First, studies will be stratified into subgroups according to distinct PSED types. Subsequently, meta-regression models will be applied to perform further subgroup analyses incorporating variations in 1) forms of TCM interventions, 2) TCM syndrome patterns, 3) treatment durations, or 4) outcome data types, among other factors. These analyses will quantify inter-subgroup differences and test their statistical significance.

#### 2.3.10 Network meta-analysis

Given that the transitivity assumption holds, we will conduct subgroup-specific random-effects network meta-analyses for PSED subtypes and TCM syndrome patterns (where data volume permits) using the “gemtc” package (version 1.0.2) in R 4.3.1 software under a Bayesian framework with non-informative priors. The NMA model will account for treatment effect correlations in multi-arm trials, comparing acupuncture, moxibustion, tuina, Chinese herbal medicine, traditional mind-body exercises (e.g., Tai Chi, Qigong), and other TCM therapies. Results will be presented in tables showing pairwise effect sizes between all interventions within each subgroup alongside probability rankings for optimal interventions (including mean ranks, 95% credible intervals, and surface under the cumulative ranking curve values). Model convergence will be evaluated using the Gelman-Rubin statistic, with values <1.1 indicating adequate convergence (50, 51).

#### 2.3.11 Sensitivity, heterogeneity, inconsistency, and evidence quality assessment

The robustness of findings will be verified by testing different priors and comparing fixed-effect versus random-effects models to exclude influences from key assumptions or data selections. Given the heterogeneity arising from inconsistent definitions of the total clinical effective rate, we will construct a primary analysis model and a sensitivity analysis model and compare the consistency of effect estimates and intervention rankings between the two models to test the robustness of the results concerning this outcome measure. If substantial differences are observed, they will be highlighted in the results and discussion sections.

According to Cochrane guidelines, I² and τ² statistics will be used to assess heterogeneity. If significant statistical heterogeneity or clinical imbalance in effect modifiers is identified, meta-regression analysis will be conducted to explore potential sources. The pre-specified effect modifiers will serve as candidate covariates. Random-effects meta-regression models will be used to examine the associations between these continuous or categorical covariates and the treatment effect size. This analysis will first be conducted within direct comparisons that have a sufficient number of studies. If the network structure and data permit, we may also employ network meta-regression within a Bayesian framework to evaluate the influence of covariates on the relative effects across the entire network.Publication bias, small-study effects, and related uncertainties will be detected and quantified through funnel plots and Egger’s regression. Since included studies may contain both two-arm and multi-arm trials, design inconsistency and loop inconsistency will be evaluated using the design-by-treatment interaction model; if inconsistency is detected within the network, closed loops will undergo further examination (52). The certainty of evidence for treatment effect estimates derived from the network meta-analysis will be assessed using a four-step approach specifically designed for NMA (53, 54). First, all direct comparisons within the network will be rated using the five standard GRADE domains. Second, for indirect comparisons, the certainty will be based on the assessment of the two contributing direct comparisons, with a focus on evaluating transitivity by comparing the distribution of potential effect modifiers across the trials forming the indirect comparison. If clinically important imbalances that threaten transitivity are identified, the evidence will be downgraded for indirectness. Third, for the mixed evidence derived from the NMA model, the assessment will start from the higher certainty rating between the available direct and indirect evidence. Network inconsistency will then be evaluated globally using the design-by-treatment interaction model. If significant and unexplained inconsistency is detected, downgrading will be applied. Finally, the certainty of evidence for each comparison will be categorized as high, moderate, low, or very low. Pre-specified operational downgrading criteria are as follows: For imprecision, downgrading will be considered if the 95% credible interval crosses a pre-defined minimal important difference or spans both the null value and a clinically important effect. For inconsistency, downgrading will be considered if evidence from direct comparisons shows a heterogeneity indicator I² > 50% with non-overlapping confidence intervals across studies, or if the P-value from network inconsistency tests is < 0.05. For indirectness, a clinically relevant imbalance in the distribution of key effect modifiers among the trials underlying an indirect comparison will serve as a basis for downgrading. Given that traditional rank probabilities based on any non-zero effect difference may be fragile and could overstate clinical superiority (54), a sensitivity analysis will be conducted to test the robustness of treatment rankings. This involves setting a series of thresholds representing minimal clinically important differences (e.g., for continuous outcomes: SMD = 0.2, 0.5, 0.8; for dichotomous outcomes: RR = 0.9, 0.8, 0.7) and recalculating the probability of each intervention being optimal. This analysis will evaluate the robustness of ranking results against clinical significance criteria.

## 3 Discussion

Stroke, ranked as the second leading cause of death globally and the third cause of mortality and disability worldwide (55), imposes a compound burden due to its high disability rate coupled with comorbid emotional disorders. Approximately 33%-50% of survivors develop affective disorders such as depression and anxiety (56). PSED severely impair patients’ quality of life across psychological, functional, and social adaptation dimensions, creating a vicious cycle with brain injury that doubles the risk of stroke recurrence and mortality. Consequently, early identification and intervention are critical for disrupting this cycle and improving prognosis. Currently, TCM demonstrates unique and significant advantages in managing PSED. By establishing a multidimensional regulatory network targeting “neuro-immuno-metabolic” pathways, TCM addresses the complex interplay of mechanisms underlying PSED through multi-target interventions (57, 58). Early TCM interventions not only effectively halt disease progression but also play a vital preventive role, substantially enhancing patient outcomes. Research indicates that early acupuncture treatment accelerates angiogenesis, promotes neuronal regeneration, and effectively inhibits apoptosis (59). For patients unable to tolerate herbal decoctions, non-pharmacological therapies such as acupuncture and Five-Element Music Therapy offer greater clinical flexibility and safety as viable alternatives. Furthermore, guided by TCM syndrome differentiation theory, treatments can be precisely tailored to match individual symptom profiles, ensuring targeted therapeutic efficacy. Finally, surveys reveal that TCM interventions provide superior cost-effectiveness ratios compared to conventional Western treatments, with patients reporting higher satisfaction levels post-treatment (60).

However, the very characteristics of TCM—diverse therapeutic approaches, multi-target regulation, and syndrome differentiation-based treatment—render clinical research in this field exceptionally complex and lacking in large-scale randomized controlled trials. Although TCM has demonstrated promising outcomes in certain disease areas, these factors impede the depth of evidence-based research on TCM interventions. Network meta-analysis (NMA), by quantifying effect sizes and ranking probabilities across multiple TCM interventions, can provide clinicians with a hierarchical selection basis for “acupuncture-Chinese herbal medicine-integrated therapy,” thereby filling the evidence gap in clinical decision-making. Nevertheless, previous TCM NMAs have predominantly focused on comparing different interventions. Given the distinctive nature of TCM syndrome differentiation and treatment, clinical studies on PSED involve varied approaches to syndrome-specific treatment. Prior TCM NMAs have not addressed efficacy comparisons across different syndrome patterns. This protocol proposes a more refined NMA design that incorporates both TCM interventions and syndrome patterns, thereby addressing the previous neglect of syndrome-specific analysis in TCM NMAs. Although this protocol proposes subgroup analysis based on TCM syndrome patterns with the aim of exploring evidence for “treating the same disease with different methods,” it also faces methodological challenges. First, heterogeneity in diagnosis and incomplete reporting are major obstacles. Despite establishing reference criteria, the included RCTs may employ different syndrome differentiation systems or report patterns only cursorily, which could lead to classification errors and residual confounding. Second, the fragmentation of sample sizes may result in a limited number of studies and patients within each syndrome subgroup, thereby reducing statistical power and making it difficult to detect true subgroup effects. These factors imply that TCM syndrome patterns are more likely to serve as effect modifiers in this NMA, rather than as absolute criteria capable of clearly delineating patient subgroups. Consequently, the results of the subgroup analysis should be interpreted as exploratory and hypothesis-generating, not as confirmatory conclusions. Nonetheless, conducting this analysis is crucial, as it represents a necessary attempt to integrate the individualized treatment philosophy of TCM into the population-based framework of evidence synthesis. Regardless of whether the results show significant differences between syndromes, this study will provide critical baseline evidence and methodological reference for future, more rigorously designed clinical trials aimed at validating the efficacy advantage of “syndrome differentiation and treatment”. Concurrently, publishing this protocol holds significance in constructing a complete chain of evidence-based research. Pre-registration compels researchers to lock the PICO elements from the outset, preventing outcome-driven analysis. Protocol registration ensures traceability in the evidence generation process, significantly reducing the risk of the “file drawer effect” (61). This process inherently adheres to the FAIR data principles, establishing an internationally compliant evidence pool for TCM’s complex intervention system. It offers direction and reference for future researchers conducting other TCM NMAs. The adoption of more rigorous evidence-based methodologies will further enhance TCM’s persuasiveness within the internationally recognized evidence hierarchy, facilitating the integration of traditional medicine achievements into global stroke management guidelines.

### 3.1 Prospects

As TCM techniques continue to be explored, developed, and refined in the future, clinical research on TCM for PSED will become more detailed, robust, and comprehensive. With the conduct of more high-quality clinical studies and the expansion of data volume, subsequent quantitative analysis via NMA will enable the identification of the most suitable TCM treatment regimens for specific syndrome patterns based on TCM characteristics. This approach will maintain the advantages of TCM’s holistic view and syndrome differentiation-based treatment while constructing an internationally recognized evidence system. Ultimately, this will achieve the goal of “articulating TCM efficacy in modern scientific terms,” promoting TCM’s greater contribution within the global health system.
